# Primary Renal Neuroendocrine Tumor Presenting as Ectopic ACTH Syndrome

**DOI:** 10.1210/jcemcr/luaf092

**Published:** 2025-04-23

**Authors:** Abrar Ali Chhachhar, Saira Furqan, Aisha Memon, Hammad Ather, Najmul Islam

**Affiliations:** Section of Diabetes, Endocrinology, and Metabolism, Department of Medicine, Aga Khan University Hospital, Karachi 74800, Pakistan; Department of Pathology & Laboratory Medicine, Aga Khan University Hospital, Karachi 74800, Pakistan; Department of Pathology & Laboratory Medicine, Aga Khan University Hospital, Karachi 74800, Pakistan; Department of Pathology & Laboratory Medicine, Aga Khan University Hospital, Karachi 74800, Pakistan; Department of Pathology & Laboratory Medicine, Aga Khan University Hospital, Karachi 74800, Pakistan

**Keywords:** ectopic ACTH syndrome, EAS, paraneoplastic Cushing syndrome, PNCS, Cushing syndrome, neuroendocrine tumor, renal-NETs

## Abstract

Ectopic adrenocorticotropin syndrome (EAS) is rarely caused by genitourinary neuroendocrine tumors (NETs). We describe an unusual case of renal NET presenting with severe EAS. A 32-year-old woman had presented to endocrine clinic with a 2-month history of proximal muscle weakness, skin hyperpigmentation, amenorrhea, and weight gain. She was hypertensive (160/90 mm Hg) with facial puffiness, hirsutism, and obesity. Investigations suggested high 8 Am cortisol, 24-hour urine free cortisol, and high adrenocorticotropin level. She required inpatient admission because of worsening symptoms, and work-up revealed severe hypokalemia and hyperglycemia requiring intravenous (IV) potassium chloride and IV insulin. Radiology revealed normal sella on magnetic resonance imaging and a mass in the upper pole of the right kidney along with bilateral adrenal enlargement on computed tomography. The patient underwent surgical resection of the right renal mass. The initial histopathology revealed renal oncocytoma, with no evidence of renal cell carcinoma, which was revisited with additional immunochemistry. Final histopathology confirmed primary renal well-differentiated NET. The abruptness and severity of symptoms favored aggressive neoplasm but histopathology confirmed an extremely rare example of primary renal NET and ruled out malignancy. This case highlights the presentation of severe EAS in a patient with extremely rare renal NET.

## Introduction

Ectopic adrenocorticotropin syndrome (EAS), referred to as paraneoplastic Cushing syndrome (PNCS), is a rare cause of adrenocorticotropin (ACTH)-dependent Cushing syndrome (CS) [1]. EAS/PNCS accounts for only 5% to 20% of all cases of endogenous CS [1, 2]. It is associated with intense hypercortisolism, resulting in severe, often life-threatening, clinical manifestations [1, 3]. EAS/PNCS is characterized by unregulated ACTH production from an extrapituitary neuroendocrine tumor (NET) of various organs, with the majority, about 90%, encountered in the respiratory and digestive tract [4]. Genitourinary NETs including renal NETs are extremely rare, and the co-occurrence of CS is even more exceptional (<1%), with fewer than 10 cases reported in the literature [5-8]. Due to the scarcity of these cases, their clinical behavior remains undetermined. Herein, we describe a rare case of primary renal well-differentiated NET presenting with EAS/PNCS and describe its key clinical features, imaging characteristics, histopathological findings, and treatment outcome.

## Case Presentation

A 32-year-old woman, with no prior comorbidities, presented to our institute with the chief complaints of proximal upper- and lower-limb weakness and skin hyperpigmentation diffusely spread over her face, neck, chest, abdomen, and back. Her symptoms started 2 months ago and rapidly progressed. She initially noticed a lack of strength while climbing upstairs and standing. Additionally, she reported hirsutism, acne, amenorrhea, and undocumented weight gain during the same period. She was recently diagnosed with diabetes mellitus and hypertension (HTN) around 1 month back.

On examination, she appeared overweight with central obesity, extensive dark pigmentation over the aforementioned regions, facial puffiness, and terminal hair growth. Her systemic examination was otherwise unremarkable except for decreased power in the proximal muscles. She did not have abdominal violaceous striae, moon facies, or a dorsocervical fat pad. Baseline work-up revealed a hemoglobin level of 11.7 g/dL (normal: 12-14.5 g/dL), a white blood cell count of 10.8 × 10^9^/L (normal: 4.0-11.0 × 10^9^/L), with a differential comprising 88.3% neutrophils (normal: 40%-70%), 6.8% lymphocytes (normal: 20%-40%), 0.1% eosinophils (normal: 1%-4%), 4.6% monocytes (normal: 2%-8%), platelet count of 261 × 10^9^/L (normal: 150-450 × 10^9^/L), serum potassium was 2.1 mmol/L (2.1 mEq/L) (normal: 3.5-5.1 mmol/L; 3.5-5.1 mEq/L), and serum bicarbonate was 39.1 mmol/L (39.1 mEq/L) (normal: 20-31 mmol/L; 20-31 mEq/L). On further work-up, 8 Am serum cortisol was greater than 60 µg/dL (>1655 nmol/L) (normal: 4.3-22.4 µg/dL; 118-618 nmol/L). While awaiting her work-up, her symptoms worsened, and she developed a new onset blurring of vision. She was referred to our emergency department.

## Diagnostic Assessment

On arrival, her blood pressure at arrival was 160/90 mm Hg and blood glucose was 375 mg/dL. The patient was admitted to a special care unit and started on continuous intravenous (IV) infusion of potassium chloride (KCL) at 8 mEq/hour and insulin infusion as per hospital protocol for hypokalemia and hyperglycemia, respectively. She required regular antihypertensive medications for elevated blood pressure. Her visual symptoms promptly improved after IV insulin and antihypertensive medications. Hypercortisolism work-up revealed her 8 Am cortisol was 103 µg/dL (2841 nmol/L) (normal: 4.3-22.4 µg/dL; 118-618 nmol/L) and 24-hour urinary free cortisol (UFC) was 15 184 µg/24 hours (41 907 nmol/24 hours) (normal: 3.5-45 µg/24 hours; 96.6-1244.2 nmol/24 hours) and significantly raised serum ACTH of greater than 1250 pg/mL (>275 pmol/L) (normal: < 46.0 pg/mL; < 10.0 pmol/L), suggesting ACTH-dependent hypercortisolism. Serum aldosterone and plasma renin were found to be normal. Her screening for pheochromocytoma was negative.

**Figure 1.**
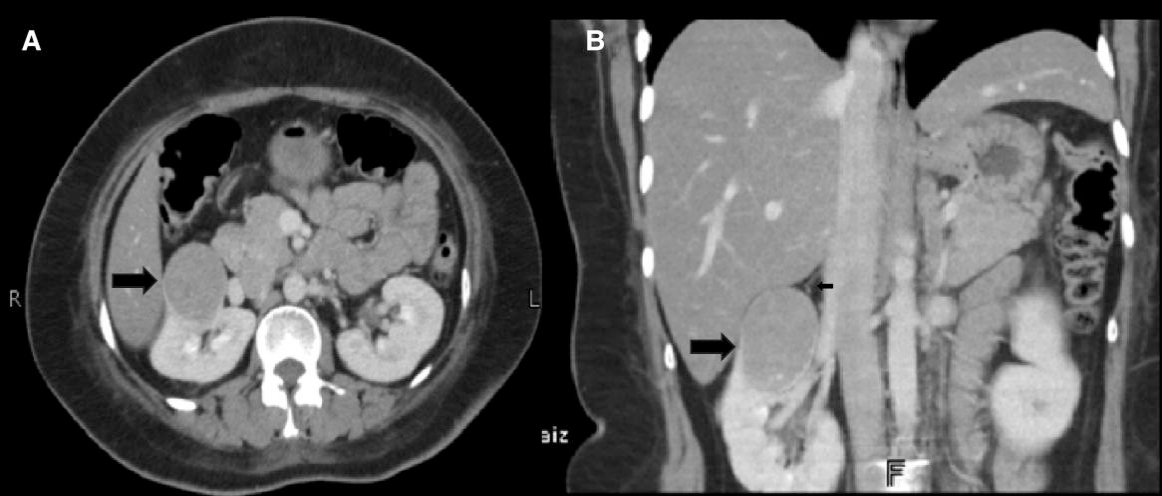
A 4-cm, round, well-circumscribed mass located in the upper pole of the right kidney, distinct from the adrenal gland on A, axial and B, coronal computed tomography of the abdomen.

In view of the above laboratory values, magnetic resonance imaging (MRI) of the pituitary revealed normal sella, and thereafter, computed tomography (CT) of the chest, abdomen, and pelvis was performed to look for a possible ectopic source. It revealed diffuse enlargement of bilateral adrenal glands with no focal lesion, suggestive of bilateral adrenal hyperplasia with an incidental finding of a round, well-circumscribed, 4-cm mass in the upper pole of the right kidney, separate from adrenal gland (Fig. 1).

## Treatment

**Figure 2.**
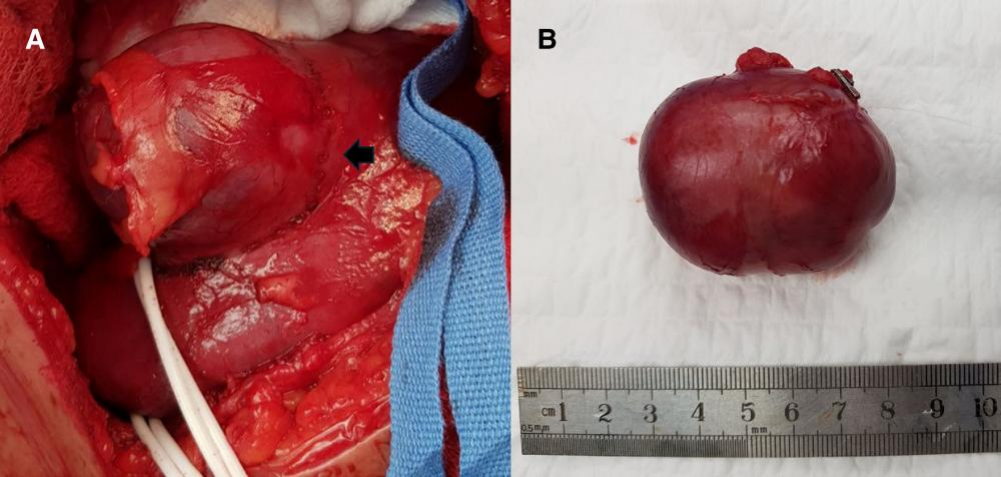
A, Preoperative and B, postoperative image shows the tumor is well demarcated with no invasion into the surrounding structures.

The patient had refractory hypokalemia that we struggled to correct despite gradually progressing IV KCL to 16 mEq/hour and very high insulin requirements. A provisional diagnosis of EAS secondary to renal cell carcinoma (RCC) was considered. Inferior petrosal sinus sampling (IPSS) could not be performed because of limited expertise. The corticotropin-releasing hormone (CRH) or desmopressin stimulation test was not pursued considering the rapid onset of severe hypercortisolism with markedly elevated ACTH levels and UFC levels along with clear radiological evidence of a renal tumor with a normal pituitary gland. Together, these findings strongly suggested an ectopic source of ACTH. Additionally, logistical challenges in arranging CRH testing in our setup, coupled with the severity of the patient's clinical condition and metabolic derangements, further influenced the decision to forgo these provocative tests and an early intervention was prioritized. She underwent a right partial nephrectomy with strict intraoperative monitoring, where the mass in its entirety was removed, while keeping clear of the renal vessels and the ipsilateral adrenal gland. The tumor was well demarcated and did not invade the surrounding structures (Fig. 2).

## Outcome and Follow-up

**Figure 3.**
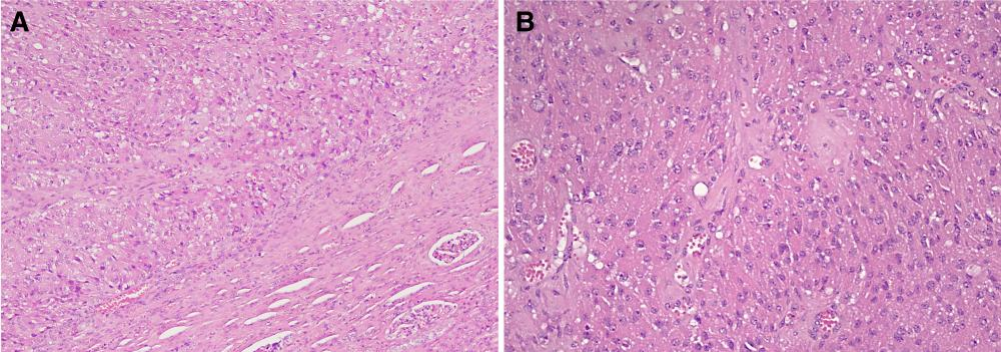
Low-power image showing intrarenal fairly circumscribed epithelial tumor with uniform polygonal cells arranged in sheets and nests with compressed renal parenchyma at periphery with hematoxylin-eosin (H&E) stain (A, ×20). High-power image showing polygonal cells with abundant granular eosinophilic cytoplasm with no significant atypia or mitoses with H&E stain (B, ×40).

**Figure 4.**
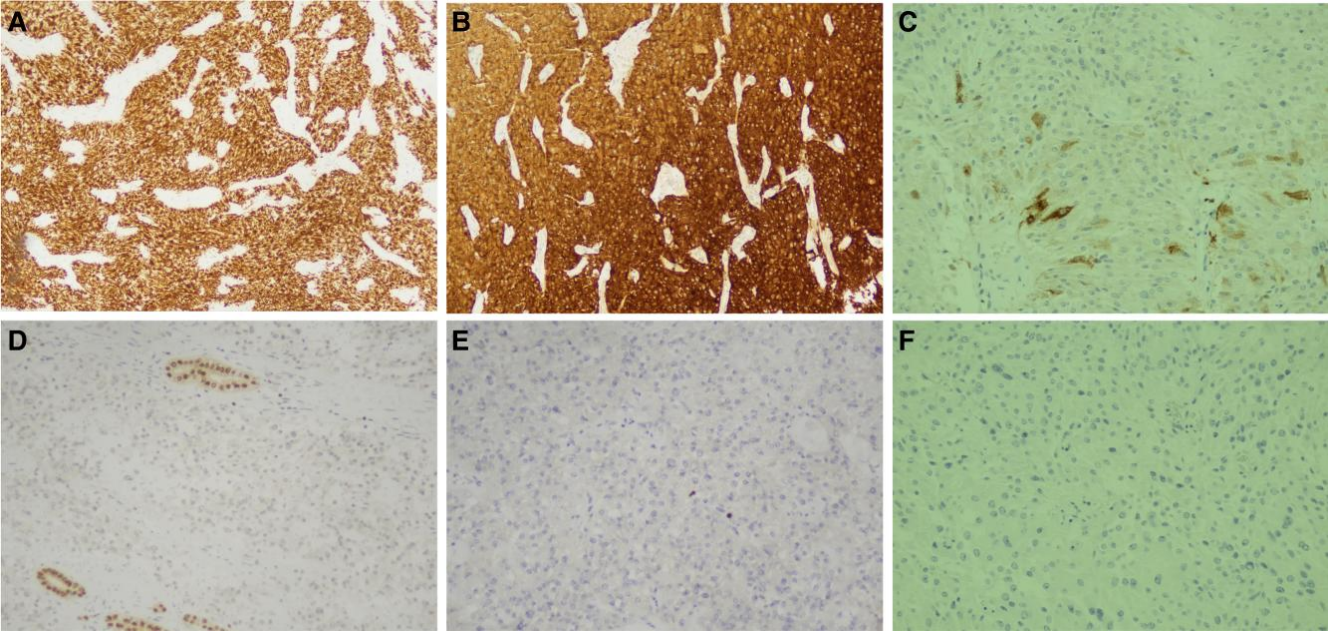
A, Cytokeratin CAM 5.2 and B, synaptophysin shows strong positivity in tumor cells. C, Chromogranin A shows weak focal positivity in tumor cells. D, Pax-8 is negative in tumor cells and highlights few entrapped renal tubules. E, Ki-67 shows a very low proliferative index (<1%). F, Inhibin is negative in tumor cells.

With an uneventful postoperative course, the patient’s blood glucose and potassium levels returned to baseline immediately after surgery, with tapering of their respective treatments. Her antihypertensive medications stopped 24 hours after surgery. An 8 Am cortisol level obtained 48 hours after surgery turned out to be 9.4 μg/dL (preoperatively >100 μg/dL). It is pertinent to mention, while we were wary of an adrenal crisis following surgery, no such event occurred postoperatively. She was discharged in stable condition. The final histopathology of the renal mass, to our surprise, did not show any component of RCC. There were nests and aggregates of round to polygonal cells with abundant eosinophilic cytoplasm and round to oval nuclei with evenly dispersed chromatin without any papillary pattern, clearing, or necrosis. Immunohistochemistry was positive for cytokeratin CAM 5.2 and CD10 but negative for vimentin and cytokeratin-7, and an initial histopathological diagnosis of renal oncocytoma was suspected. However, it was revisited, and additional immunochemical stains were performed that turned out to be positive for synaptophysin, neuron-specific enolase, and focal positivity for chromogranin A. Immunostaining for ACTH was not available in our institution. A final diagnosis of ACTH-secreting, primary renal well-differentiated NET was made (Figs. 3 and 4).

The patient reported substantial improvement in her acne and skin hyperpigmentation within 2 months postoperatively. Her menstrual cycles became regular and she experienced considerable improvement in muscle weakness and was able to resume her normal work activities. She was able to discontinue her diabetic and HTN medications. Given the possibility of residual or recurrent hypercortisolism, the patient was advised to undergo repeat electrolytes, 8 Am cortisol, and ACTH testing along with imaging 3 to 6 months postoperatively. However, the patient, who was not a native of our country, left the area and was subsequently lost to follow-up.

## Discussion

Renal NETs are rare tumors and account for less than 1% of all NETs [8]. Paraneoplastic syndromes caused by renal NETs are even more uncommon, and only 4% of all NETs are associated with EAS/PNCS. It is followed by carcinoid syndrome and a single reported case of insulinoma and gastrinoma, each associated with EAS [7]. This case illustrates several important challenges in the diagnosis and management of EAS caused by renal NET. EAS, associated with very high serum cortisol and ACTH levels, presents with rapid onset and severe hypercortisolism as in this case. Our patient presented with abrupt onset of proximal myopathy, body swelling with diffuse skin pigmentation, menstrual irregularities, hypokalemia, metabolic alkalosis, newly diagnosed diabetes mellitus, and HTN. The patient required inpatient care to treat hyperglycemia and refractory hypokalemia with insulin infusion and IV potassium replacement, respectively. Biochemistry confirming markedly raised serum and UFC level along with ACTH made EAS the very likely diagnosis.

To establish the cause of EAS requires cross-sectional imaging modalities such as CT or positron emission tomography (PET) with CT (PET/CT) of the chest, abdomen, and pelvis to localize the tumor. Recently several functional imaging modalities, such as Octreoscan or the new gallium-68 labeled somatostatin receptor PET/CT (68Ga-SSTR PET/CT), have shown encouraging results in EAS/PNCS [3]. Even though it is not available in our country, in our case, the cause of EAS/PNCS syndrome was found to be a round, well-circumscribed, 4-cm mass at the upper pole of right kidney, with all other imaging being normal. Because of the abruptness and severity of symptoms, a provisional diagnosis of EAS secondary to RCC, an aggressive tumor, was made as radiologically it cannot be differentiated from renal NET [9]. It was substantiated after metabolic derangements reverted to normal instantaneously and improvement in clinical features after the surgical removal of the tumor, confirming the source of the EAS.

Postoperative hypocortisolism, or tertiary adrenal insufficiency, is seen in all subtypes of CS following surgical removal of the underlying tumor, requiring glucocorticoid replacement. This insufficiency occurs due to chronic glucocorticoid excess, which suppresses CRH-producing cells and impairs hypothalamic-pituitary-adrenal axis function. And the recovery times vary depending on the underlying etiology. It is longest in adrenal CS caused by a unilateral cortisol-producing adenoma, intermediate in Cushing disease, and shortest in EAS. In our case, to our surprise, our patient did not encounter postoperative hypocortisolism. This may be attributed to the hypothalamic-pituitary-adrenal axis not being suppressed, potentially because of the acute onset of the illness and rapid initiation of treatment, similar to the findings of Berr et al [10]. However, the patient was provided with education on the sick day rules for hydrocortisone use, considering the 48-hour postoperative serum cortisol level of 9.4 µg/dL.

After successful surgical removal of the tumor, initial histopathological diagnosis was renal oncocytoma. After thorough literature review, we came across 5 rare instances in which the oncocytoma was found to be associated with paraneoplastic manifestations. Pertinent to mention, none of these included CS. Based on all this information, the histopathology was revisited, additional immunochemical stains were performed, and the tumor was found to be positive for synaptophysin and neuron-specific enolase. It showed focal positivity for chromogranin A. A final diagnosis of ACTH-secreting primary renal well-differentiated NET was made and consistent with the histopathologic findings of oncocytic renal NET reported by Hannah et al and Kasajima et al [5, 7]. However, while chromogranin A was diffusely expressed in CS-associated renal NETs, as reported by Kasajima et al [7], chromogranin A showed focal weak positivity in our case. Although ACTH immunostaining was not available, the diagnosis of EAS secondary to a renal NET was strongly supported by the patient's clinical presentation, biochemical markers, and radiological findings of a renal mass, along with rapid recovery following tumor removal.

Our patient did well after surgical excision of the tumor, the primary mode of therapy for EAS patients. In our patient’s case, clinical improvement and the initial postoperative workup pointed toward the improvement of her EAS after source removal. Although the follow-up period was limited to 8 weeks, long-term monitoring with ACTH and 8 Am cortisol is needed to ensure the resolution of ACTH-dependent CS and to detect any potential recurrence. We recommend continued surveillance in such cases due to their rarity, as prognosis and clinical behavior remain unclear.

## Learning Points

- EAS, given its high morbidity and mortality, must be suspected in patients with abrupt onset and severe hypercortisolism.
- The source of occult EAS should be pursued as per standard endocrinology guidelines, including MRI of the brain, thin-slice CT imaging of the whole body, and IPSS.
- IPSS may not be performed in EAS due to the severity of the clinical condition and associated risk.
- The only curative option for EAS is the surgical resection or elimination of the ACTH-secreting neoplasm.
- Renal NETs, difficult to distinguish from RCC radiologically, require keen awareness and discrete use of immunohistochemical neuroendocrine markers.

## Contributors

All authors made individual contributions to authorship. A.A., S.F., and N.I. were involved in the diagnosis and management of the patient and manuscript submission. A.M. was involved in the histopathology section and preparation of histology images. H.A. was responsible for the patient's surgeries. All authors reviewed and approved the final draft.

## Data Availability

Data sharing is not applicable to this article as no data sets were generated or analyzed during the present study.

## Abbreviations

ACTH: adrenocorticotropin
CRH: corticotropin-releasing hormone
CS: Cushing syndrome
CT: computed tomography
EAS: ectopic adrenocorticotropin syndrome
HTN: hypertension
IPSS: inferior petrosal sinus sampling
IV: intravenous
KCL: potassium chloride
MRI: magnetic resonance imaging
NET: neuroendocrine tumor
PET: positron emission tomography
PNCS: paraneoplastic Cushing syndrome
RCC: renal cell carcinoma
UFC: urinary free cortisol
